# ATG16L1 meets ATG9 in recycling endosomes: Additional roles for the plasma membrane and endocytosis in autophagosome biogenesis

**DOI:** 10.4161/auto.27174

**Published:** 2013-11-15

**Authors:** Claudia Puri, Maurizio Renna, Carla Figueira Bento, Kevin Moreau, David C Rubinsztein

**Affiliations:** Department of Medical Genetics; Cambridge Institute for Medical Research; University of Cambridge; Cambridge UK

**Keywords:** autophagy, endocytosis, ATG16L1, mATG9, recycling endosome, VAMP3

## Abstract

Autophagosomes are formed by double-membraned structures, which engulf portions of cytoplasm. Autophagosomes ultimately fuse with lysosomes, where their contents are degraded. The origin of the autophagosome membrane may involve different sources, such as mitochondria, Golgi, endoplasmic reticulum, plasma membrane, and recycling endosomes. We recently observed that ATG9 localizes on the plasma membrane in clathrin-coated structures and is internalized following a classical endocytic pathway through early and then recycling endosomes. By contrast, ATG16L1 is also internalized by clathrin-mediated endocytosis but via different clathrin-coated pits, and appears to follow a different route to the recycling endosomes. The R-SNARE VAMP3 mediates the coalescence of the 2 different pools of vesicles (containing ATG16L1 or ATG9) in recycling endosomes. The heterotypic fusion between ATG16L1- and ATG9-containing vesicles strongly correlates with subsequent autophagosome formation. Thus, ATG9 and ATG16L1 both traffic from the plasma membrane to autophagic precursor structures and provide 2 routes from the plasma membrane to autophagosomes.

Macroautophagy, hereafter referred to as autophagy, is a membrane-mediated process that delivers cytoplasmic materials to lysosomes for degradation. Activation of autophagy leads to the formation of cup-shaped structures (phagophores), which elongate and seal to become double-membraned autophagosomes that have engulfed portions of the cytoplasm. Later, autophagosomes fuse with lysosomes and the engulfed material is degraded.

Multiple membrane sources have been proposed for autophagosomes, including the endoplasmic reticulum, mitochondria, plasma membrane, the Golgi, and recycling endosomes. However, the events that precede phagophore assembly are still quite unclear. We recently demonstrated that plasma membrane contributes to phagophore/autophagosome assembly. The autophagic protein ATG16L1 is internalized by clathrin-mediated endocytosis and undergoes homotypic fusion events that precede phagophore assembly and LC3 acquisition. We wanted to understand if these ATG16L1-containing vesicles (which also contain the associated members of the ATG12–ATG5 complex) met other ATG proteins prior to phagophore assembly, and focused on ATG9. Because it is a multipass transmembrane protein, one assumes that ATG9 will carry membranes with it from the compartments through which it traffics. While ATG9 is required for optimal autophagosome biogenesis and is associated with mammalian autophagosomes, true fusion with autophagosome-related structures had not been reported in mammalian cells, although previous studies had not looked at ATG16L1-positive phagophore precursors.

We found that ATG9 trafficks through the plasma membrane and is internalized by clathrin-mediated endocytosis. After internalization, ATG9 is delivered to recycling endosomes via a conventional route through early endosomes, following the canonical transferrin receptor internalization pathway. Interestingly, the ATG16L1 and ATG9 are found in distinct clathrin-coated pits. Furthermore, these proteins appear to follow distinct paths to recycling endosomes, since there is no obvious localization of ATG16L1 in early endosomes (Fig. 1).

**Figure:**
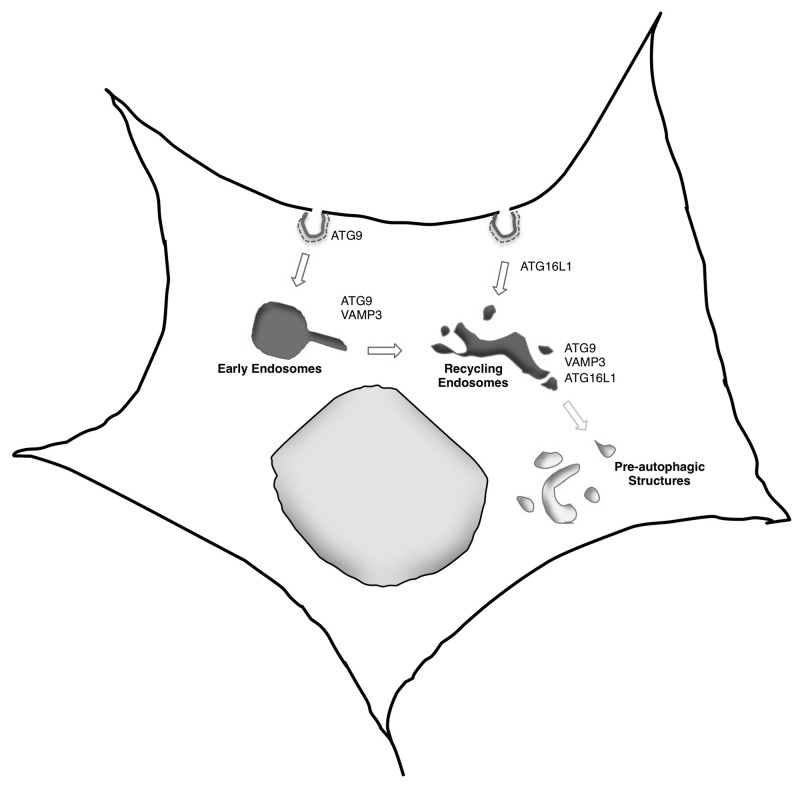
**Figure 1.** Schematic diagram of ATG9 and ATG16L1 trafficking pathways, showing how they meet and fuse in a VAMP3-dependent manner in recycling endosomes.

Since we found both ATG16L1 and ATG9 in recycling endosomes, we tested and confirmed that vesicles containing these 2 ATG proteins fuse in this compartment. The observation that both ATG9 and ATG16L1 colocalize in transferrin-positive, RAB11-positive, but LC3-negative, structures, led us to speculate that the heterotypic fusion of the 2 pools of vesicles could represent an early event of phagophore assembly. Indeed, lowering the temperature to 18 °C, which leads to a block of trafficking from early endosomes to recycling endosomes, impairs the fusion between ATG16L1- and ATG9-containing vesicles, and inhibits autophagosome formation. While this temperature block is a nonspecific approach, we strengthened the hypothesis with a complementary strategy, by reducing the egress from the recycling endosomes by overexpressing either RAB11 or the MYO5B/myosin Vb tail (dominant negative). In this scenario, the fusion between ATG9- and ATG16L1-containing vesicles increases and autophagy is induced. Interestingly, a similar phenomenon occurs in cells exposed to starvation, a primordial autophagy stimulus; in these conditions, recycling is slowed and there is enhanced ATG9-ATG16L1 vesicle fusion.

Intracellular vesicular fusions are generally SNARE dependent. We tested and confirmed that this was the case for ATG16L1-ATG9 vesicle fusion, since N-ethylmaleimide, which inhibits SNARE activity, reduces the colocalization between ATG16L1 and ATG9 and impairs in vitro fusion of vesicles derived from postnuclear supernatant fractions containing either ATG9 or ATG16L1. Generally, SNARE complexes are made up of 3 molecules with highly conserved glutamine (Q) residues in the SNARE motif, and 1 with a highly conserved arginine (R) residue in this domain; these are referred to as Qa-, Qb-, Qc-, and R-SNAREs. In order to identify relevant SNAREs, we performed an siRNA-based screen for R-SNAREs affecting the levels of the autophagy substrate SQSTM1/p62 and found that VAMP3 knockdown elevates SQSTM1 levels and decreases autophagosome formation. This SNARE was an attractive candidate to consider as a regulator of ATG9-ATG16L1 vesicle fusion, as it localizes on early and recycling endosomes. VAMP3 depletion leads to an accumulation of ATG9 in early endosomes and ATG16L1 in recycling endosomes, but does not affect the general endocytic pathway (since endocytosis of TFR/transferrin receptor and recycling endosome morphology are normal). VAMP3 also appears to traffic with ATG9 from early to recycling endosomes (Fig. 1). VAMP3 knockdown strongly affects the fusion of ATG9- and ATG16L1-containing vesicles both in cells and in vitro.

Together, our data suggest that the plasma membrane contributes to autophagosome formation through at least 2 distinct ATG proteins, ATG9 (which routes to the recycling endosomes through the early endosomal compartment), and ATG16L1 (which is transported from the plasma membrane to the recycling endosomes, bypassing the early endosomal compartment). Notably, these 2 pathways are already completely separated at the plasma membrane, as the clathrin-coated structures that are carrying the 2 autophagic proteins are distinct, but then converge in the recycling endosomes. The SNARE VAMP3 is co-trafficked with ATG9, and its activity is critical for ATG9-ATG16L1 vesicle fusion and autophagosome biogenesis.

These studies shed new light on the functions of recycling endosomes. Until now, this compartment was just thought to be involved in the recycling of several plasma membrane receptors back to the plasma membrane. Here we show that this compartment is also important for some early steps of autophagosome formation. Indeed, our data suggest that recycling endosome membrane is incorporated into ATG16L1-containing vesicles. Finally, these observations raise some new questions, such as: Why does the plasma membrane contribute to autophagosome biogenesis by trafficking via 2 distinct routes? How are ATG9 and ATG16L1 partitioned into different clathrin-coated structures? Perhaps most importantly, these data raise the possibility that membrane-trafficking events and fusions between distinct compartments initially containing different ATG proteins can occur prior to the phagophore stage (since the ATG9-ATG16L1 vesicle fusions occur in LC3-negative structures). Thus, it may be prudent to look upstream of the phagophore stage for clues about the origins of autophagosome membranes and some of the factors regulating autophagosome biogenesis.

## Notes

10.4161/auto.27174

